# Physical exercise and glycemic management in patients with type 1 diabetes on insulin pump therapy—a cross-sectional study

**DOI:** 10.1007/s00592-023-02070-7

**Published:** 2023-03-24

**Authors:** Margarida Ferreira, João Sérgio Neves, Celestino Neves, Davide Carvalho

**Affiliations:** 1 Faculty of Medicine of Medicine of the University of Porto, Porto, Portugal; 2 Department of Endocrinology, Diabetes and Metabolism, Centro Hospitalar Universitário de São João, EPE, Porto, Portugal; 3 Cardiovascular Research and Development Center, Department of Surgery and Physiology, Faculty of Medicine, University of Porto, Porto, Portugal; 4 Instituto de Investigação E Inovação Em Saúde, University of Porto, Porto, Portugal

**Keywords:** Type 1 diabetes, Insulin pump, Physical exercise, Continuous glucose monitor, Time in Range

## Abstract

**Aims:**

Exercise is an important practice for control in type 1 diabetes (T1D). This study aims to assess de association between exercise and glycemic management in people with T1D and to identify the main barriers to exercise in T1D.

**Methods:**

We evaluated 95 people with T1D treated with insulin pump therapy. Participants answered a questionnaire about 1) exercise habits, 2) usual adjustments in insulin and food intake with exercise and 3) main barriers to exercise.

Continuous glucose monitoring (CGM) was used to evaluate time in range (TIR), time below range (TBR) and time above range (TAR) during the last 60 days before the evaluation. CGM data during, before (2 h before) and after (24 h after) the last bout of exercise was also evaluated.

**Results:**

The mean age was 30.1 ± 12.1 years, and 51.6% were women. Participants that reported practicing exercise (55.8%) had a higher TIR (59.6 ± 16.3 vs. 48.7 ± 15.7, *p* = 0.012) and a lower TAR (32.6 ± 15.8 vs. 45.4 ± 17.7, *p* = 0.006). Comparing with the 60 days CGM data, the TBR was lower in the 2 h before exercise (− 1.8 ± 3.8, *p* = 0.0454) and TAR was lower during (− 16.9 ± 33.6, *p* = 0.0320) and in the 24 h after (− 8.7 ± 17.2, *p* = 0.032) the last bout of exercise. The absence of adjustments on insulin and food intake was associated with higher TBR after the exercise (13.44 ± 3.5, *p* < 0.05). Eating before the exercise and turning off the pump during the exercise were associated with lower TBR after exercise (food booster: − 7.56 ± 3.49, *p* < 0.05; turning off insulin pump − 8.87 ± 3.52, *p* < 0.05). The main barriers reported for exercise practicing were fear of hypoglycemia, lack of free time and work schedule.

**Conclusion:**

Exercise was associated with better glycemic management in people with T1D. Addressing common barriers may allow a higher adherence to exercise in T1D.

**Supplementary Information:**

The online version contains supplementary material available at 10.1007/s00592-023-02070-7.

## Introduction

Type 1 diabetes (T1D) is a chronic disease that usually presents early in life and impairs the patient’s glucose metabolism [1]. Its incidence and prevalence are rising in the world, with 15 new cases per 100,000 people and a more than 9.000.000 people living with T1D worldwide [2, 3].

The management of the disease is complex causing a high burden in the people with T1D daily life and affecting adhesion to treatment. Consequently, a high proportion of them doesn’t achieve the intended targets and are at higher risk of developing complications and of having early mortality [4, 5].

The challenges in glycemic management may lead to hyperglycemia and diabetic ketoacidosis, the most important acute complication of this disease, or to hypoglycemia, which is frequently related to physical exercise [6]. As the disease progresses, the cardiovascular morbimortality increases. People with T1D may also have other cardiovascular risk factors, which may further increase the cardiovascular risk—in people with T1D, and sedentarism and obesity are central risk factors for dyslipidemia, hypertension, insulin resistance, and elevated inflammatory markers [7, 8].

The treatment of T1D consists of daily insulin administration for life, through multiple daily injections or insulin pump [9], dietary control and physical exercise [10].

Exercise recommendations for adults with T1D are at least 150 cumulative minutes of exercise per week, with no more than 2 consecutive days without exercise. Regular physical exercise has the potential to improve cardiovascular risk factors, blood glucose and decrease the risk of complications, improving the quality of life and longevity of people with T1D [6]. However, people with T1D are at least as sedentary as the rest of the population and most of them do not achieve this target [11, 12].

Challenges associated with exercise that are specific to T1D, such as glycemic management and fear of hypoglycemia, contribute to the low compliance with the recommendations [12, 13]. Also, if insulin and food intake are not correctly adjusted to physical activity, exercise may contribute to increased risk of both hypoglycemia and hyperglycemia [14].

This occurs due to the central role of carbohydrate metabolism in the exercise physiology. In people without T1D, there is a decrease in insulin secretion and an increase in glucagon, growth hormone, cortisol and catecholamine secretion in response to exercise [15]. These mechanisms maintain the blood glucose concentrations in a tight range (~ 4–6 mmol/l; ~ 70–100 mg/dl) [11]. In people with T1D, several of these processes are affected. Since insulin is exogenous, its levels aren’t reduced with exercise and might even increase due to increased absorption, redistribution, and higher rate of blood flow [11]. Simultaneously, the release of counterregulatory hormones is reduced in T1D during exercise [16].

The response to different types of exercise also differs and is affected in T1D. Aerobic exercise stimulates glucose uptake by the muscles. High insulin levels enhance this uptake and suppress endogenous glucose production, which leads to hypoglycemia. On the other hand, anaerobic exercise and high-intensity interval training (HIIT) cause an important adrenergic activation that stimulates endogenous glucose production. In people with T1D, the body can’t increase insulin production in response, which leads to hyperglycemia [11, 16].

After aerobic exercise, glucose usage remains elevated to replenish its storage, leading to a high risk of hypoglycemia in the 24 h after exercise. In anaerobic exercise and in HIIT, hyperglycemia may persist after exercise and its correction needs to be done with caution in order to avoid hypoglycemia [11, 16].

These variations make glucose control in exercise practice a challenge. Thus, this study aims to assess the association between physical exercise and glycemic management in people with T1D on insulin pump therapy, and to identify the main barriers they find to exercise. We hypothesized that participants that usually perform physical exercise have better glycemic management.

## Methods

### Study design and subjects

This cross-sectional study targeted outpatients from Centro Hospitalar Universitário de São João (CHUSJ) diagnosed with type 1 diabetes who were on insulin pump therapy at the time of recruitment and were at least 18 years old. They were included in the study regardless of complications. The participants were recruited between December 2021 and February 2022. All people with T1D and insulin pump therapy were eligible to participate in this study.

The questionnaire and methodology for this study were approved by the hospital’s ethics comm﻿ittee and all participants signed an informed consent, after reciving the information about the study and its implications.

### Demographic and clinical data

Data were collected from the medical records: 1) demographic data, consisting of age, sex, occupation and age at diagnosis; 2) clinical data, consisting of glycosylated hemoglobin (HbA1c), main microvascular (retinopathy, nephropathy, and peripheral neuropathy) and macrovascular complications (coronary disease, cerebrovascular disease), mean total daily insulin dose, basal insulin, insulin bolus, insulin carbohydrate ratio (ICR) and insulin sensitivity factor (ISF), height, weight, waist circumference, arterial tension and lipid profile.

Body mass index (BMI) was calculated as weight in kilograms divided by height in meters squared. Lipid profile consisted in total cholesterol, high-density lipoprotein and low-density lipoprotein cholesterol and triglycerides levels from the last biochemical analysis.

When the participants had a FreeStyle Libre continuous glucose monitor (CGM), time in range (TIR), time below range (TBR), time above range (TAR), average glucose and coefficient of variation in the last 60 days were recorded. Time in target was considered from 70 to 180 mg/dL and hypoglycemia and hyperglycemia below and above that range, respectively [17].

### Questionnaire and CGM data

A two-part questionnaire was developed and applied.

The first question inquired if the patient practiced exercise. If the answer was negative, they could skip to the second part. If the answer was affirmative, the first part of the questionnaire went on to characterize physical exercise in frequency, type (aerobic, anaerobic and high-intensity interval training (HIIT)) and duration. Participants were asked to describe the adjustments in basal insulin, insulin bolus and food intake they usually made before, during and up to 24 h after exercise. It also asked the number of times of exercise in the previous 7 days, as well as day, time, type and duration of the last bout of practice.

In the participants who had FreeStyle Libre glucose monitor, this information was then used to quantify the TIR, TBR and TAR in following periods: 1) the 2 h before the exercise, 2) during the exercise and 3) during the 24 h after exercise. The glucose level at the beginning of the last bout of exercise (normoglycemia, hypoglycemia or hyperglycemia) was also collected.

The second part of the questionnaire was directed to all the participants and consisted of a list of barriers to physical exercise which they had to classify based on how much it affected them (from 1—not a barrier, to 4—decisive barrier). This list was based on the BAPAD-1 scale and on previous studies on barriers to exercise in people with T1D [12, 18].

### Statistical analysis

The comparison of continuous variables between participants with or without exercise practice was performed with independent t tests. For comparisons within the same participant of the TIR, TBR and TAR before, during and after the exercise (vs. the 60-day period) we used paired t tests. Differences between groups regarding categorical variables were evaluated with Chi-squared test. To evaluate the effect of adjustments in basal insulin, insulin bolus and food intake, we used linear regression models. The correlation of TIR, TBR and TAR with the questionnaire on the barriers to exercise was analyzed by the Pearson correlation test. The normality of the data was evaluated with the visual evaluation of the histogram. Results are presented as mean ± standard deviation for continuous variables and as percentages for categorical variables. A two-sided *P*-value of < 0.05 was considered statistically significant. All analyses were performed using Stata® (StataCorp. 2021. Stata Statistical Software: Release 17. College Station, TX: StataCorp LLC).

## Results

### Demographic and clinical data

Two hundred and twenty-three adults with T1D on insulin pump therapy are followed in CHUSJ. One hundred and four of them were invited to participate, and 9 refused to participate.

The final sample consisted of 95 adults, 46 of which were men (48.4%) and 49 women (51.6%), with a mean age of 30.1 ± 12.1 years and 12.5 ± 9.3 years at diagnosis. The participants used stand-alone insulin pumps for a mean time of 6 years, either Medtronic Paradigm Veo or Accu-Chek Combo Insulin Pump. They had a mean BMI of 24.6 ± 3.7 kg/m^2^ and abdominal circumference of 83.4 ± 13.3 cm. The mean HbA1C was 7.5 ± 1.0% (58 mmol/mol). Fourteen participants had T1D complications at the time of recruitment, 22 retinopathy (23.2%), 18 nephropathy (18.9%), 3 neuropathy (3.2%) and 4 coronary artery disease (4.2%). None had cerebrovascular disease. These weren’t significantly different between those who exercised and those who didn’t. All participants using FreeStyle Libre used version 2. Sample study characteristics are shown in Table 1, as well as a subdivision in groups according to exercise status. Thirty-one participants had missing data on CGM parameters (13 participants were not using CGM, 2 were using alternative systems that did not allow the collection of the data; 6 patients had data on TIR but not data on TBR or TAR). In the last 60 days, mean TIR was 55.6 ± 16.8%, TBR was 5.2 ± 6.6%, and TAR was 37.8 ± 17.7%.

**Table 1:** Participant characteristics (*n* = 95)

Variable	Category	All participants (*n* = 95)	No exercise (*n* = 42)	Exercise (*n* = 53)	
Age (years)		30.1 ± 12.1	29.7 ± 11.6	30.4 ± 12.5	*p* = 0.79
Sex	Male	46 (48.4%)	17 (40.5%)	29 (54.7%)	*p* = 0.17
	Female	49 (51.6%)	25 (59.5%)	24 (45.3%)	
Profession	Administration	3 (3.4%)	3 (7.5%)	0 (0.0%)	*p* = 0.21
	Commercial	2 (2.3%)	1 (2.5%)	1 (2.1%)	
	Management	7 (8.9%)	3 (7.5%)	4 (8.3%)	
	Production/crafts/industry	3 (3.4%)	0 (0.0%)	3 (6.3%)	
	Scientist/Specialist/arts	23 (26.1%)	9 (22.5%)	14 (29.2%)	
	Services	16 (18.2%)	10 (25.0%)	6 (12.5%)	
	Student	31 (35.2%)	14 (35.0%)	17 (35.4%)	
	Retired	1 (1.1%)	0 (0.0%)	1 (2.1%)	
	Unemployed	2 (2.3%)	0 (0.0%)	2 (4.2%)	
BMI (kg/m^2^)		24.6 ± 3.7	25.6 ± 3.9	24.6 ± 3.6	*p* = 0.95
Abdominal circumference (cm)		83.4 ± 13.2	81.9 ± 13.3	84.5 ± 13.2	*p* = 0.39
Age at diagnosis (years)		12.5 ± 9.3	11.6 ± 9.7	13.2 ± 8.9	*p* = 0.38
Complications	Retinopathy	22 (23.2%)	10 (23.8%)	12 (22.6%)	*p* = 0.89
	Nephropathy	18 (18.9%)	7 (16.7%)	11 (20.8%)	*p* = 0.61
	Neuropathy	3 (3.2%)	0 (0.0%)	3 (5.7%)	*p* = 0.12
	Coronary disease	4 (4.2%)	3 (7.1%)	1 (1.9%)	*p* = 0.21
	Cerebrovascular disease	0 (0%)	0 (0.0%)	0 (0.0%)	–
Insulin pump data	ICR (g/U)	10.2 ± 3.1	10.0 ± 2.8	10.4 ± 3.3	*p* = 0.55
	ISF	41.9 ± 17.7	39.9 ± 15.4	43.4 ± 19.2	*p* = 0.34
	Total daily insulin (U)	51.1 ± 18.4	51.6 ± 16.0	50.6 ± 20.3	*p* = 0.79
	Daily basal (U)	25.4 ± 9.2	26.5 ± 8.3	24.5 ± 9.9	*p* = 0.29
	Daily Bolus (U)	25.6 ± 12.0	25.1 ± 10.7	26.0 ± 13.0	*p* = 0.74
HbA1c (%) (mmol/mol)		7.5 ± 1.0 (58)	7.6 ± 1.0 (60)	7.4 ± 1.0 (57)	*p* = 0.20
SAP (mmHg)		126.0 ± 12.4	126.6 ± 11.5	125.5 ± 13.2	*p* = 0.68
DAP (mmHg)		72.9 ± 10.3	75.7 ± 8.9	70.7 ± 10.9	*p* = 0.019
Total cholesterol (mg/dl)		167.7 ± 34.0	166.8 ± 36.4	168.4 ± 32.4	*p* = 0.83
HDL (mg/dl)		58.0 ± 15.3	55.7 ± 15.6	59.8 ± 15.0	*p* = 0.22
LDL (mg/dl)		93.8 ± 26.1	95.0 ± 28.7	93.0 ± 24.0	*p* = 0.72
Triglycerides (mg/dl)		78.9 ± 37.8	83.5 ± 43.9	75.2 ± 32.2	*p* = 0.31
FreeStyle Libre (glucose monitor; last 60 days)	TIR (%)	55.6 ± 16.8	48.7 ± 15.7	59.6 ± 16.3	*p* = 0.012
	TAR (%)	37.8 ± 17.6	45.4 ± 17.7	32.6 ± 15.8	*p* = 0.006
	TBR (%)	5.2 ± 6.6	6.0 ± 8.0	4.7 ± 5.4	*p* = 0.50
	Mean glucose (mg/dl)	170.9 ± 35.6	185.1 ± 42.0	161.0 ± 27.0	*p* = 0.011
	Glucose variability (%)	39.9 ± 7.5	41.5 ± 8.1	38.9 ± 6.9	*p* = 0.20
Physical exercise	Yes	53 (55.8%)			
	No	42 (44.2%)			
	Times per week	1–2			16 (30.2%)
		3–4			22 (41.5%)
		≥ 5			15 (28.3%)
	Hours per week	< 1			2 (3.8%)
		1–2			16 (30.2%)
		> 2			35 (66.0%)
	Type of exercise	Aerobic			25 (47.2%)
		Aerobic and anaerobic			22 (41.5%)
		Aerobic, anaerobic and HIIT			5 (9.4%)
		Anaerobic			1 (1.9%)

Data presented are *n* (%) mean ± SD

*BMI* body mass index, *DBP* diastolic blood pressure, *HbA1c* glycosylated hemoglobin, *HDL* high-density cholesterol, *HIIT* high-intensity interval training, *ICR* Insulin carbohydrate ratio, *ISF* insulin sensitivity factor, *LDL* low-density cholesterol, *SBP* systolic blood pressure, *TAR* time above range, *TBR* time below range, *TIR* time in range

### Physical exercise

Fifty-three participants (55.8%) practiced exercise: 25 (47.2%) aerobic, 22 (41.5%) aerobic and anaerobic, 5 aerobic, anaerobic and HIIT, and 1 only anaerobic. Sixteen (30.2%) participants exercised up to two times per week, 22 (41.5%) three or four times a week and 15 (28.3%) five or more. 35 (66.0%) of these participants exercised more than 2 h per week (Table 1).

When comparing the two subgroups, TIR was significantly higher in those who practice exercise and TAR, mean glucose and diastolic arterial pressure were significantly lower.

Table 2 characterizes the last exercise done by the participants, describes TIR, TAR and TBR in the 2 h before, during the exercise and in the 24 h after the exercise, and compares these parameters with mean values over the last 60 days. TBR in the 2 h before exercise and TAR during and up to 24 h after exercise are significantly lower than the TBR and TAR mean CGM times, respectively.

**Table 2:** Characterization of last exercise practice and glycemic variation (using GCM data)

			Difference with mean values from the last 60 days﻿	*p* value
Duration of exercise (hours)	1.3 ± 0.7			
Type of exercise	Aerobic	14 (66.7%)		
	Aerobic and anaerobic	3 (14.3%)		
	Anaerobic	4 (19.0%)		
Beginning of exercise	With glycemia within TIR n (%)	17 (81.0%)		
	With glycemia within TBR n (%)	0 (0.0%)		
	With glycemia within TAR n (%)	4 (19.0%)		
Before exercise (2 h)	TIR (%)	76.2 ± 37.4	13.3 ± 36.0	0.1061
	TBR (%)	1.8 ± 5.0	− 1.8 ± 3.8	0.0454
	TAR (%)	22.2 ± 38.1	− 11.5 ± 37.2	0.1700
During exercise	TIR (%)	73.4 ± 39.2	10.5 ± 35.5	0.1914
	TBR (%)	8.4 ± 22.5	5.0 ± 22.6	0.3185
	TAR (%)	16.9 ± 35.6	− 16.9 ± 33.6	0.0320
After exercise (24 h)	TIR (%)	65.1 ± 18.8	2.2 ± 12.3	0.4183
	TBR (%)	5.7 ± 7.9	2.4 ± 8.2	0.2023
	TAR (%)	25.1 ± 20.9	− 8.7 ± 17.2	0.0320

*CGM* continuous glucose monitor, *TAR* time above range, *TBR* time below range, *TIR* time in range

### Adjustments to insulin and food intake and glycemic variation with exercise

The adjustments made by the participants to insulin and food intake are detailed in Table 3. The most frequent adjustment is eating extra food before exercise, usually carbohydrates according to the participants, which is done by 22 participants (41.5%). Six participants (11.3%) do not make any adjustments to exercise on food or insulin.

**Table 3:** Insulin and food intake adjustments to exercise

Time	Adjustments	*n* = 53
Meal before exercise	Reduce insulin bolus	11 (20.8%)
	No insulin bolus	1 (1.9%)
	Reduce carbohydrates	1 (1.9%)
Before exercise	Reduce basal insulin	7 (13.2%)
	Extra insulin bolus	3 (5.7%)
	Exercise after meal	4 (7.5%)
	Food booster	22 (41.5%)
During exercise	Turn off insulin pump	14 (26.4%)
	Reduce basal insulin	8 (15.1%)
After exercise	Reduce basal insulin	15 (28.3%)
	Increase basal insulin	1 (1.9%)
	Food booster	13 (24.5%)
Meal after exercise	Reduce insulin bolus	5 (9.4%)
No adjustments		6 (11.3%)

The association of the adjustments to insulin and food intake with CGM data before, during and after the exercise is described in supplementary Table 1. Participants who ate extra food before exercise (supplementary Table 3) and those who turned off the insulin pump (supplementary Table 4) during exercise had a significantly lower TBR in the 24 h after exercise, when compared to the mean CGM TBR. Participants who made no adjustments in insulin or food intake had a significantly higher TBR (supplementary Table 5).

### Questionnaire—barriers to exercise

‘The participants’ classification of barriers to exercise according to importance is described in Table 4. Fear of hypoglycemic episodes, trouble with work schedule and lack of free time were the most important barriers.

**Table 4:** Questionnaire: barriers to physical exercise

Barrier	Degree of importance	All patients	Mean score ± SD
Lack of information on how to adjust insulin and food intake to exercise	1	59 (62.1%)	1.6 ± 0.9
	2	16 (16.8%)	
	3	16 (16.8%)	
	4	4 (4.2%)	
Fear of worsening glycemic control	1	52 (54.7%)	1.8 ± 1.0
	2	21 (22.1%)	
	3	13 (13.7%)	
	4	9 (9.5%)	
Fear of hypoglycemic episodes	1	21 (22.1%)	2.5 ± 1.1
	2	25 (26.3%)	
	3	25 (26.3%)	
	4	24 (25.3%)	
Fear of hyperglycemic episodes	1	46 (48.4%)	1.8 ± 0.9
	2	29 (30.5%)	
	3	13 (13.7%)	
	4	7 (7.4%)	
Fear of feeling tired	1	61 (64.2%)	1.6 ± 0.9
	2	20 (21.1%)	
	3	7 (7.4%)	
	4	7 (7.4%)	
Low fitness level	1	47 (49.5%)	1.8 ± 0.9
	2	27 (28.4%)	
	3	16 (16.8%)	
	4	5 (5.3%)	
Not knowing what to do	1	68 (71.6%)	1.4 ± 0.8
	2	16 (16.8%)	
	3	7 (7.4%)	
	4	4 (4.2%)	
Fear of getting injured	1	61 (64.2%)	1.5 ± 0.8
	2	21 (22.1%)	
	3	9 (9.5%)	
	4	4 (4.2%)	
Lack of company	1	62 (65.3%)	1.6 ± 1.0
	2	14 (14.7%)	
	3	12 (12.6%)	
	4	7 (7.4%)	
Lack of support	1	84 (88.4%)	1.2 ± 0.6
	2	2 (2.1%)	
	3	8 (8.4%)	
	4	1 (1.1%)	
Trouble with the work schedule	1	30 (31.6%)	2.4 ± 1.2
	2	18 (18.9%)	
	3	23 (24.2%)	
	4	24 (25.3%)	
Lack of free time	1	26 (27.4%)	2.5 ± 1.2
	2	18 (18.9%)	
	3	26 (27.4%)	
	4	25 (26.3%)	
Lack of conditions	1	57 (60.0%)	1.7 ± 0.9
	2	18 (18.9%)	
	3	15 (15.8%)	
	4	5 (5.3%)	

When comparing participants who exercise to those who do not, fear of worsening glycemic management, lack of company to exercise, trouble with work schedule and lack of free time are significantly more important to those who do not don’t exercise (supplementary Table 1).

Other barriers described by participants were: knee injury (1 patient), psychological fatigue related to disease burden (1), dumping syndrome (1), covid pandemic (2), tiredness hard to recover from after hypoglycemia during exercise (1) and pump related, physical discomfort (2), carrying the pump (1) and accessories needed (1).

We analyzed the correlation between the questionnaire on barriers to exercise and TIR, TBR and TAR. We found that there was a negative correlation between the score on the questionnaire and TIR (*r* = − 0.147, *p* = 0.008), and a positive correlation with TAR (*r* = 0.131, *p* = 0.016). TBR was not significantly correlated with the score on the questionnaire (*r* = 0.155, *p* = 0.296).

## Conclusions

In this cross-sectional study, we observed that exercise improved glucose levels. Participants who practice exercise had higher TIR and lower TAR, suggesting a beneficial effect of exercise on glycemic management. There was a decrease of TAR during and after the last bout of exercise compared to the mean TAR in the last 60 days, showing a direct beneficial effect of exercise on preventing hyperglycemia. Different profiles of usual adjustments to food intake and insulin were associated with an impact on the TBR after exercise. The most reported barriers to exercise were fear of hypoglycemia, trouble with work schedule and lack of free time.

Exercise is an important part of T1D treatment with great potential to help in glycemic management, cardiovascular risk and overall health and wellbeing. However, it simultaneously poses a threat to disease control if the patient doesn’t know the appropriate management [16]. Therefore, it’s important to evaluate usual exercise practice, the main barriers to exercise and the association between exercise and glycemic management, using this information to improve patient care.

Despite the increase in TIR in those who practice exercise, the difference of mean HbA1c between groups (7.4 ± 1.0% (57 mmol/mol) in those who exercise vs. 7.6 ± 1.0% (60 mmol/mol) in those who do not exercise) was not statistically significant, similarly to what was described in previous studies [19]. This might be related to inconsistent exercise practice, decrease in insulin and increase in carbohydrate intake around exercise and overall difficulty in exercise management, resulting in less effect on glycemia in the long term. Even so, the observed difference in TIR (+ 10.9% in those who exercise) is probably clinically relevant. TIR is correlated with diabetes complications, is less affected by other medical conditions and is a good tool to guide interventions [20].

Although several previous studies evaluated the association of exercise with glycemic management, few studies evaluated the association of usual adjustments in insulin and food intake to exercise with glycemic management during and after exercise.

Our results highlight the need of interventions to prevent hypoglycemia, as participants who do not make any changes have higher TBR. Simultaneously, food intake before exercise and suspending the insulin pump during exercise were associated with lower TBR in the 24 h after the exercise, protecting against hypoglycemia.

Our study also reinforces the importance of adequate adjustments on food intake and insulin therapy, to allow better glycemic management during and after exercise, and it is consistent with the recommendations from the recent consensus on exercise management on T1D [11, 21].

Accordingly, if aerobic exercise is planned and happens 2–3 h after a meal, reducing the meal’s insulin bolus is a good strategy to avoid hypoglycemia. Reducing the basal insulin after exercise reduces the risk of late hypoglycemia. An alternative that also reduces the risk of hypoglycemia during exercise is suspending basal insulin during exercise, as shown in our study, but it has a higher risk of hyperglycemia after the exercise [11].

When exercise is not planned and/or changes in insulin were not made, food intake is an important tool to prevent hypoglycemia. If glucose levels before exercise are below 7.0 mmol/l (127 mg/dl), carbohydrates might be consumed to reduce this risk, especially for prolonged aerobic exercise. During exercise, glucose targets are between 5.0 and 10.0 mmol/l (90–180 mg/dl). If glucose levels get close to the lower threshold during exercise, carbohydrates should be consumed during exercise to avoid hypoglycemia [21].

For anaerobic exercise or HIIT, there is a higher risk of hyperglycemia, so reduction of inulin or food intake before exercise is not usually recommended. These adjustments might be necessary after exercise to prevent late hypoglycemia, but only if hyperglycemia has not developed [11].

Given the lack of normal physiologic response to exercise in T1D and the complexity of its management, teaching people with T1D about the glycemic variations with exercise and the role of insulin and food intake adjustments can prevent hypoglycemia, which is one of their main barriers to exercise, and is essential to optimize control and make this management easier [22]. Data from CGM might be a useful tool for people with T1D to learn the best adjustments for exercise, since it reflects short-term glucose variations [20].

Fear of hypoglycemic episodes was one of the main barriers to exercise reported, in agreement with several previous studies [12, 23, 24]. Adequate education on strategies to prevent hypoglycemia related to exercise may be essential to allow a higher number of people with T1D to adhere to exercise guidelines.

It is interesting to note, however, that a recent study demonstrated that greater knowledge about the management of T1D and physical activity was not associated with less hypoglycemia during and after physical [25].

Participants who do not exercise, compared to those who exercise, reported higher scores of fear of worsening glycemic management with exercise. Specific education on exercise practice for sedentary people with T1D may allow an increase in the perception of the benefits of exercise in T1D for disease control and increase the confidence to manage glucose variations, consequently increasing patient adherence to physical activity.

Lack of company to exercise, trouble with work schedule and lack of free time were also more reported by those who do not exercise. This highlights the importance of helping people with T1D to find strategies and solutions not only for barriers related to T1D, but also for barriers that are present in the general population.

Our study has some limitations. Since this was a cross-sectional study, we cannot establish a causal relationship between the significant associations described. Since our population was restricted to a single center, and to people with T1D with insulin pump, the generalization of our findings to other populations must be done with caution. It is also important to note that the information on exercise habits was self-reported, with the possibility of not representing the real practice. Additionally, we only evaluated TBR and TAR as global values and not according to TBR or TAR level 1 and 2.

In conclusion, physical exercise was associated with better glycemic management in people with T1D treated with insulin pumps. Improving adjustments on food intake and insulin therapy with exercise may decrease the risk of hypoglycemia after exercise. Addressing common barriers to exercise practice in T1D may be important to increase the adherence to exercise.

## Supplementary Information

Below is the link to the electronic supplementary material.

Supplementary file1 (DOCX 32 KB)

## Data Availability

The datasets generated during and/or analyzed during the current study are available from the corresponding author on reasonable request.

## References

[1] Eizirik DL, Pasquali L, Cnop M (2020). Pancreatic β-cells in type 1 and type 2 diabetes mellitus: different pathways to failure. Nat Rev Endocrinol.

[2] Mobasseri M, Shirmohammadi M, Amiri T, Vahed N, Hosseini Fard H, Ghojazadeh M (2020). Prevalence and incidence of type 1 diabetes in the world: a systematic review and meta-analysis. Health promot perspect.

[3] Green A, Hede SM, Patterson CC, Wild SH, Imperatore G, Roglic G, Beran D (2021). Type 1 diabetes in 2017: global estimates of incident and prevalent cases in children and adults. Diabetologia.

[4] Pérez-Marín M, Gómez-Rico I, Montoya-Castilla I (2015). Type 1 diabetes mellitus: psychosocial factors and adjustment of pediatric patient and his/her family. Rev Archivos argentinos de pediatria.

[5] Gonzalez JS, Tanenbaum ML, Commissariat PV (2016). Psychosocial factors in medication adherence and diabetes self-management: implications for research and practice. Am Psychol.

[6] Lu X, Zhao C (2020). Exercise and Type 1 Diabetes. Adv Exp Med Biol.

[7] Snell-Bergeon JK, Nadeau K (2012). Cardiovascular disease risk in young people with type 1 diabetes. J Cardiovasc Transl Res.

[8] Redondo MJ, Foster NC, Libman IM, Mehta SN, Hathway JM, Bethin KE, Nathan BM, Ecker MA, Shah AC, DuBose SN, Tamborlane WV, Hoffman RP, Wong JC, Maahs DM, Beck RW, DiMeglio LA (2016). Prevalence of cardiovascular risk factors in youth with type 1 diabetes and elevated body mass index. Acta Diabetol.

[9] Nathan, D. M., & DCCT/EDIC Research Group (2014). The diabetes control and complications trial/epidemiology of diabetes interventions and complications study at 30 years: overview. Diabetes Care.

[10] Atkinson MA, Eisenbarth GS, Michels AW (2014). Type 1 diabetes. Lancet (London, England).

[11] Riddell MC, Gallen IW, Smart CE (2017). Exercise management in type 1 diabetes: a consensus statement. Lancet Diabetes Endocrinol.

[12] Brazeau AS, Rabasa-Lhoret R, Strychar I, Mircescu H (2008). Barriers to physical activity among patients with type 1 diabetes. Diabetes Care.

[13] Chetty T, Shetty V, Fournier PA, Adolfsson P, Jones TW, Davis EA (2019). Exercise management for young people with type 1 diabetes: a structured approach to the exercise consultation. Front Endocrinol.

[14] van Dijk JW, Eijsvogels TM, Nyakayiru J, Schreuder TH, Hopman MT, Thijssen DH, van Loon LJ (2016). Glycemic control during consecutive days with prolonged walking exercise in individuals with type 1 diabetes mellitus. Diabetes Res Clin Pract.

[15] Riddell MC, Perkins BA (2006). Type 1 diabetes and vigorous exercise: applications of exercise physiology to patient management. Can J Diabetes.

[16] Galassetti P, Riddell MC (2013). Exercise and type 1 diabetes (T1DM). Compr Physiol.

[17] American Diabetes Association (2021). 6 Glycemic targets: standards of medical care in diabetes—2021. Diabetes Care.

[18] Brazeau AS, Mircescu H, Desjardins K, Dubé MC, Weisnagel SJ, Lavoie C, Rabasa-Lhoret R (2012). The barriers to physical activity in type 1 diabetes (bapad-1) scale: predictive validity and reliability. Diabetes Metab.

[19] Kennedy A, Nirantharakumar K, Chimen M, Pang TT, Hemming K, Andrews RC, Narendran P (2013). Does exercise improve glycaemic control in type 1 diabetes? A systematic review and meta-analysis. PLoS ONE.

[20] Vigersky RA, McMahon C (2019). The relationship of hemoglobin a1c to time-in-range in patients with diabetes. Diabetes Technol Ther.

[21] Moser O, Riddell MC, Eckstein ML (2020). Glucose management for exercise using continuous glucose monitoring (CGM) and intermittently scanned CGM (isCGM) systems in type 1 diabetes: position statement of the European Association for the Study of Diabetes (EASD) and of the International Society for Pediatric and Adolescent Diabetes (ISPAD) endorsed by JDRF and supported by the American Diabetes Association (ADA). Diabetologia.

[22] Drenthen L, Abbink EJ, Thijssen D, Tack CJ, de Galan BE (2021). Sporten en insulinegebruik [Physical exercise and insulin use: challenges for people with diabetes mellitus]. Ned Tijdschr Geneeskd.

[23] Andre CC, Kudva YC, Dadlani V, Cescon M, Mccrady-Spitzer SK, Church MM, Reid C, Kumari K, Choudhary D, Doyle FJ, Dassau E, Pinsker JE (2019). 734-P: Perceived Barriers to Physical Activity in People with Type 1 Diabetes Using CGM. Diabetes.

[24] Roberts AJ, Taplin CE (2015). Exercise in youth with type 1 diabetes. Curr Pediatr Rev.

[25] Paiement K, Frenette V, Wu Z, Suppère C, Messier V, Lasalle-Vaillancourt A, Mathieu ME, Rabasa-Lhoret R (2022). Is better understanding of management strategies for adults with type 1 diabetes associated with a lower risk of developing hypoglycemia during and after physical activity?. Can J Diabetes.

