# Supplementary material for: Physical exercise and glycemic management in patients with type 1 diabetes on insulin pump therapy—a cross-sectional study
Source: Acta Diabetol. 2023 Mar 24;60(7):881–9. doi: 10.1007/s00592-023-02070-7 (PMC10198918; doi:10.1007/s00592-023-02070-7)
Supplement: Supplementary file 1 — Supplementary file1 (DOCX 32 KB) [file 592_2023_2070_MOESM1_ESM.docx]

**Supplements**

Supplementary table 1 - Questionnaire: Barriers to Physical Exercise (No Exercise vs. Exercise)

| **Barrier** | **No Exercise** | **Exercise** | **p-value** |
| --- | --- | --- | --- |
| Lack of information on how to adjust insulin and food intake to exercise | 1.7 ± 0.9 | 1.6 ± 0.9 | 0.58 |
| Fear of worsening glycemic control | 2.0 ± 1.1 | 1.6 ± 0.9 | 0.020 |
| Fear of hypoglycemic episodes | 2.6 ± 1.1 | 2.5 ± 1.1 | 0.57 |
| Fear of hyperglycemic episodes | 1.9 ± 1.0 | 1.8 ± 0.9 | 0.60 |
| Fear of feeling tired | 1.7 ± 0.9 | 1.5 ± 0.9 | 0.41 |
| Low fitness level | 2.0 ± 0.9 | 1.6 ± 0.9 | 0.10 |
| Not knowing what to do | 1.6 ± 0.9 | 1.3 ± 0.7 | 0.06 |
| Fear of getting injured | 1.4 ± 0.7 | 1.7 ± 0.9 | 0.11 |
| Lack of company | 2.0 ± 1.1 | 1.4 ± 0.8 | 0.003 |
| Lack of support | 1.4 ± 0.8 | 1.1 ± 0.5 | 0.06 |
| Trouble with the work schedule | 2.9 ± 1.1 | 2.1 ± 1.1 | 0.001 |
| Lack of free time | 2.9 ± 1.0 | 2.2 ± 1.2 | 0.004 |
| Lack of conditions | 1.8 ± 1.0 | 1.6 ± 0.9 | 0.25 |

SD, standard deviation

Supplementary table 2 - Variation of continuous glucose monitor data before, during and after the exercise, according to adjustments in insulin and food intake, comparing with 60-days data.

|  |  | **Variation of CGM data before, during and after the exercise comparing with 28-days data** | | | | | | | | |
| --- | --- | --- | --- | --- | --- | --- | --- | --- | --- | --- |
|  |  | **Before (2 hours)** | | | **During** | | | **After (24 hours)** | | |
|  |  | **TIR** | **TBR** | **TAR** | **TIR** | **TBR** | **TAR** | **TIR** | **TBR** | **TAR** |
| Meal before exercise | Reduce insulin bolus | -14.61 ± 16.78 | 0.39 ± 1.79 | 14.21 ± 17.35 | 2.30 ± 16.84 | -9.07 ± 10.53 | 3.46 ± 15.92 | -10.14 ± 5.38 | -6.21 ± 3.63 | 14.79 ± 7.45 |
|  | No insulin bolus | 0 | 0 | 0 | 0 | 0 | 0 | 0 | 0 | 0 |
|  | Reduce carbohydrates | 0 | 0 | 0 | 0 | 0 | 0 | 0 | 0 | 0 |
| Before exercise | Reduce basal insulin | 31.18 ± 37.21 | 1.85 ± 3.96 | -33.03 ± 38.34 | 34.16 ± 36.47 | -5.30 ± 23.74 | -27.43 ± 34.71 | 12.93 ± 12.62 | -1.60 ± 8.63 | -22.93 ± 17.34 |
|  | Extra insulin bolus | 43.32 ± 25.63 | 0.84 ± 2.88 | -44.16 ± 26.48 | 18.82 ± 26.72 | 20.95 ± 16.56 | -38.24 ± 24.04 | -0.46 ± 9.41 | -2.49 ± 6.23 | -0.14 ± 13.14 |
|  | Exercise after meal | -9.18 ± 27.40 | -0.82 ± 2.88 | 10.00 ± 28.26 | -19.86 ± 26.68 | -8.34 ± 17.14 | 29.71 ± 24.67 | -6.14 ± 9.30 | 7.68 ± 6.01 | -15.63 ± 12.64 |
|  | Food booster | -22.86 ± 16.29 | 2.64 ± 1.69 | 20.21 ± 17.05 | -20.59 ± 16.18 | 9.36 ± 10.52 | 7.15 ± 15.85 | -1.35 ± 5.85 | -7.56 ± 3.49 * | 10.41 ± 7.83 |
| During exercise | Turn off insulin pump | 2.01 ± 17.9 | -0.22 ± 1.87 | -1.80 ± 18.42 | -24.40 ± 16.67 | -1.93 ± 11.20 | 21.58 ± 15.88 | 0.32 ± 6.11 | -8.87 ± 3.52 * | 14.26 ± 7.89 |
|  | Reduce basal insulin | 10.09 ± 18.80 | -0.84 ± 1.98 | -9.26 ± 19.43 | 3.85 ± 18.63 | 14.38 ± 11.42 | -16.43 ± 17.23 | -1.39 ± 6.47 | -1.02 ± 4.31 | -0.97 ± 9.06 |
| After exercise | Reduce basal insulin | -1.46 ± 20.54 | 0.01 ± 2.16 | 1.45 ± 21.19 | 5.90 ± 20.19 | -8.40 ± 12.75 | 4.17 ± 19.11 | -5.35 ± 6.92 | -4.40 ± 4.57 | 10.54 ± 9.52 |
|  | Increase basal insulin | 28.03 ± 37.34 | -1.30 ± 3.97 | -26.73 ± 38.60 | 31.01 ± 36.62 | -8.45 ± 23.69 | -21.13 ± 34.94 | 21.42 ± 12.00 | -4.89 ± 8.56 | -12.25 ± 17.90 |
|  | Food booster | 10.12 ± 20.42 | -1.84 ± 2.12 | -8.28 ± 21.11 | 22.89 ± 19.54 | 11.37 ± 12.62 | -32.58 ± 17.61 | -7.61 ± 6.81 | -5.00 ± 4.54 | 14.42 ± 9.25 |
| Meal after exercise | Reduce insulin bolus | 11.97 ± 20.36 | 1.10 ± 2.14 | -13.07 ± 20.98 | 16.71 ± 19.86 | -12.41 ± 12.57 | -10.34 ± 18.98 | -6.56 ± 6.87 | -5.89 ± 4.48 | 17.46 ± 8.97 |
| No adjustments | | -9.96 ± 20.42 | -1.84 ± 2.12 | 11.79 ± 21.02 | 1.17 ± 20.23 | -5.93 ± 12.82 | 6.44 ± 19.07 | 6.85 ± 6.85 | 13.44 ± 3.52 * | -17.82 ± 8.93 |

* p<0.05

CGM, continuous glucose monitor; TAR, time above range; TBR, time below range; TIR, time in range.

Supplementary table 3 - Comparison of CGM data between patients that eat something before exercise (vs. those that do not eat something before exercise).

|  | |  | **No food booster before exercise (n=14)** | **Food booster before exercise (n=7)** | **p-value** |
| --- | --- | --- | --- | --- | --- |
| Type of last exercise | | Aerobic | 10 (71.4%) | 4 (57.1%) | 0.41 |
|  |  | Aerobic and anaerobic | 1 (7.1%) | 2 (28.6%) |  |
|  |  | Anaerobic | 3 (21.4%) | 1 (14.3%) |  |
| Duration of exercise | |  | 1.2 ± 0.8 | 1.5 ± 0.6 | 0.41 |
| Mean CGM times (last 60 days) | | TIR | 66.6 ± 15.7 | 55.6 ± 12.2 | 0.12 |
|  |  | TAB | 30.8 ± 16.0 | 39.7 ± 16.1 | 0.24 |
|  |  | TBR | 2.6 ± 1.9 | 4.7 ± 4.7 | 0.16 |
| Glucose variability | |  | 36.5 ± 6.7 | 40.3 ± 4.6 | 0.20 |
| Before exercise  (2 hours) | | TIR | 87.5 ± 32.2 | 53.6 ± 39.2 | 0.47 |
|  |  | TBR | 0.00 ± 0.00 | 4.71 ± 8.05 | 0.04 |
|  |  | TAR | 12.5 ± 32.2 | 41.6 ± 43.9 | 0.10 |
| During exercise | | TIR | 83.9 ± 31.7 | 52.3 ± 46.4 | 0.08 |
|  |  | TBR | 4.6 ± 13.6 | 16.0 ± 34.3 | 0.28 |
|  |  | TAR | 11.5 ± 28.8 | 27.6 ± 47.2 | 0.34 |
| After exercise  (24 hours) | | TIR | 69.2 ± 20.4 | 56.9 ± 12.3 | 0.16 |
|  |  | TBR | 7.5 ± 9.1 | 2.0 ± 2.6 | 0.14 |
|  |  | TAR | 18.6 ± 21.6 | 38.0 ± 12.3 | 0.042 |
| Difference with mean CGM times | Before exercise  (2 hours) | TIR | 20.9 ± 30.4 | -1.9 ± 43.9 | 0.18 |
|  |  | TBR | -2.6 ± 1.9 | 0.0 ± 5.8 | 0.13 |
|  |  | TAR | -18.3 ± 30.6 | 1.9 ± 47.5 | 0.25 |
|  | During exercise | TIR | 17.3 ± 25.6 | -3.3 ± 49.5 | 0.22 |
|  |  | TBR | 1.9 ± 13.7 | 11.3 ± 35.0 | 0.38 |
|  |  | TAR | -19.3 ± 26.4 | -12.1 ± 46.9 | 0.66 |
|  | After exercise (24 hours) | TIR | 2.7 ± 11.6 | 1.3 ± 14.7 | 0.82 |
|  |  | TBR | 4.9 ± 8.6 | -2.7 ± 4.4 | 0.043 |
|  |  | TAR | -12.1 ± 17.9 | -1.7 ± 14.5 | 0.20 |

CGM, continuous glucose monitor; TAR, time above range; TBR, time below range; TIR, time in range.

Supplementary table 4 - Comparison of CGM data between patients that turn the insulin pump off during exercise (vs. those that do not turn the insulin pump off during exercise).

|  | |  | **Didn’t turn pump off (n=15)** | **Turned Pump off (n=6)** | **p-value** |
| --- | --- | --- | --- | --- | --- |
| Type of last exercise | | Aerobic | 9 (60.0%) | 5 (83.3%) | 0.37 |
|  |  | Aerobic and anaerobic | 2 (13.3%) | 1 (16.7%) |  |
|  |  | Anaerobic | 4 (27.6%) | 0 (0%) |  |
| Duration of exercise | |  | 1.3 ± 0.7 | 1.3 ± 0.8 | 0.99 |
| Mean CGM times (last 60 days) | | TIR | 63.4 ± 13.8 | 61.7 ± 19.8 | 0.82 |
|  |  | TAB | 33.8 ± 14.0 | 33.7 ± 22.5 | 0.99 |
|  |  | TBR | 2.8 ± 2.0 | 4.7 ± 5.1 | 0.23 |
| Glucose variability | |  | 37.8 ± 7.0 | 37.7 ± 4.3 | 0.99 |
| Before exercise  (2 hours) | | TIR | 76.1 ± 38.2 | 76.4 ± 38.9 | 0.99 |
|  |  | TBR | 1.10 ± 4.26 | 2.75 ± 6.74 | 0.51 |
|  |  | TAR | 22.8 ± 38.7 | 20.8 ± 40.1 | 0.92 |
| During exercise | | TIR | 80.8 ± 35.4 | 54.7 ± 45.1 | 0.17 |
|  |  | TBR | 8.4 ± 23.9 | 8.3 ± 20.4 | 1.00 |
|  |  | TAR | 10.8 ± 27.9 | 32.2 ± 49.9 | 0.22 |
| After exercise  (24 hours) | | TIR | 65.5 ± 17.9 | 64.1 ± 22.6 | 0.88 |
|  |  | TBR | 7.7 ± 8.6 | 0.7 ± 1.4 | 0.07 |
|  |  | TAR | 21.1 ± 19.2 | 35.2 ± 23.2 | 0.17 |
| Difference with mean CGM times | Before exercise  (2 hours) | TIR | 12.7 ± 34.3 | 14.8 ± 43.5 | 0.91 |
|  |  | TBR | -1.7 ± 4.4 | -1.9 ± 1.7 | 0.91 |
|  |  | TAR | -11.0 ± 35.9 | -12.8 ± 43.5 | 0.92 |
|  | During exercise | TIR | 17.4 ± 33.2 | -7.0 ± 37.8 | 0.16 |
|  |  | TBR | 5.6 ± 23.5 | 3.7 ± 22.3 | 0.86 |
|  |  | TAR | -23.0 ± 26.1 | -1.5 ± 46.8 | 0.19 |
|  | After exercise (24 hours) | TIR | 2.1 ± 11.5 | 2.5 ± 15.4 | 0.96 |
|  |  | TBR | 4.9 ± 8.2 | -4.0 ± 3.7 | 0.021 |
|  |  | TIR | -12.7 ± 16.4 | 1.5 ± 16.0 | 0.09 |
|  |  |  |  |  |  |

CGM, continuous glucose monitor; TAR, time above range; TBR, time below range; TIR, time in range.

Supplementary table 5 – Comparison of CGM data between patients that make adjustments in food intake or insulin therapy (vs. those that did not make any adjustments in food intake or insulin therapy).

|  | |  | **Adjustments (n=17)** | **No Adjustments (n=4)** | **p-value** |
| --- | --- | --- | --- | --- | --- |
| Type of last exercise | | Aerobic | 10 (58.8%) | 4 (100%) | 0.29 |
|  |  | Aerobic and anaerobic | 3 (17.6%) | 0 (0%) |  |
|  |  | Anaerobic | 4 (23.5%) | 0 (0%) |  |
| Duration of exercise | | | 1.2 ± 0.6 | 1.4 ± 1.1 | 0.63 |
| Mean CGM times (last 60 days) | | TIR | 61.3 ± 15.6 | 69.8 ± 13.1 | 0.33 |
|  |  | TAB | 35.4 ± 16.7 | 27.0 ± 13.6 | 0.37 |
|  |  | TBR | 3.4 ± 3.4 | 3.3 ± 2.4 | 0.96 |
| Glucose variability | |  | 38.2 ± 5.7 | 35.8 ± 8.8 | 0.50 |
| Before exercise  (2 hours) | | TIR | 76.5 ± 35.8 | 75.0 ± 50.0 | 0.94 |
|  |  | TBR | 1.94 ± 5.48 | 0.00 ± 0.00 | 0.50 |
|  |  | TAR | 21.6 ± 36.6 | 25.0 ± 50.0 | 0.88 |
| During exercise | | TIR | 71.5 ± 41.5 | 81.2 ± 30.6 | 0.67 |
|  |  | TBR | 9.5 ± 24.8 | 3.5 ± 7.0 | 0.64 |
|  |  | TAR | 17.2 ± 38.4 | 15..3 ± 23.7 | 0.93 |
| After exercise  (24 hours) | | TIR | 62.2 ± 18.6 | 77.5 ± 15.7 | 0.15 |
|  |  | TBR | 3.2 ± 4.8 | 16.5 ± 10.1 | < 0.001 |
|  |  | TAR | 30.1 ± 20.0 | 3.9 ± 5.7 | 0.020 |
| Difference with mean CGM times | Before exercise  (2 hours) | TIR | 15.2 ± 35.3 | 5.3 ± 43.5 | 0.63 |
|  |  | TBR | -1.4 ± 4.0 | -3.3 ± 2.4 | 0.40 |
|  |  | TAR | -13.8 ± 36.3 | -2.0 ± 44.9 | 0.58 |
|  | During exercise | TIR | 10.2 ± 38.2 | 11.4 ± 24.7 | 0.95 |
|  |  | TBR | 6.2 ± 25.0 | 0.3 ± 6.2 | 0.65 |
|  |  | TAR | -18.1 ± 36.4 | -11.7 ± 19.8 | 0.74 |
|  | After exercise (24 hours) | TIR | 0.9 ± 13.1 | 7.8 ± 6.6 | 0.33 |
|  |  | TBR | -0.2 ± 5.2 | 13.2 ± 10.5 | 0.001 |
|  |  | TAR | -5.3 ± 16.8 | -23.1 ± 11.8 | 0.06 |
|  |  |  |  |  |  |

CGM, continuous glucose monitor; TAR, time above range; TBR, time below range; TIR, time in range.

**Questionnaire**

**No.** _______

Select the answer that best applies to you.

1. **Do you practice physical exercise?** (if the answer is no, you can proceed to question 14)

Yes  No

1. **How often?**

less than 1 time per week

1 to 2 times per week

3 to 4 times per week

5 or more times per week

1. **How many hours of exercise do you do on average per week??**

less than 1 hour per week

1 to 2 hours per week

more than 2 hours per week

1. **What kind of exercise do you do?** (you can select more than 1 option)

aerobic (walking, running, cycling, swimming…)

anaerobic (strength exercises – with body weight, free weights, machines, elastic resistance bands)

HIIT (High Intensity Interval Training – combination of brief, very-high intensity bursts of cardio exercise followed by equal or longer periods of rest.)

1. **Usually, what adjustments do you make to basal insulin before, during or up to 24 hours after exercise?**

______________________________________________________________________________________________________________________________________________________

1. **Usually, what adjustments do you make to insulin bolus before, during or up to 24 hours after exercise?**

______________________________________________________________________________________________________________________________________________________

1. **Usually, what adjustments do you make in food intake before, during or up to 24 hours after exercise?**

______________________________________________________________________________________________________________________________________________________

1. **How many times did you exercise last week (last 7 days)?** ______________________
2. **When was the last time you exercised?** (day and time) __________________________________________________________________________
3. **What type of exercise did you do and for how long?**

___________________________________________________________________________

1. **In the last time you exercised, what adjustments did you make to basal insulin before, during and up to 24 hours after exercise?**

______________________________________________________________________________________________________________________________________________________

1. **In the last time you exercised, what adjustments did you make to insulin bolus before, during and up to 24 hours after exercise?**

______________________________________________________________________________________________________________________________________________________

1. **In the last time you exercised, what adjustments did you make in food intake before, during and up to 24 hours after exercise?**

______________________________________________________________________________________________________________________________________________________

1. **To what degree do you consider each of these a barrier to exercise for you?**

**1 –** It’s not a barrier.

**2 –** It’s a barrier of little importance.**3 –** It’s an important barrier.

**4 –** It’s a decisive barrier.

|  | **1** | **2** | **3** | **4** |
| --- | --- | --- | --- | --- |
| Lack of information on how to adjust insulin and food intake to exercise |  |  |  |  |
| Fear of worsening glycemic control |  |  |  |  |
| Fear of hypoglycemic episodes |  |  |  |  |
| Fear of hyperglycemic episodes |  |  |  |  |
| Fear of feeling tired |  |  |  |  |
| Low fitness level |  |  |  |  |
| Not knowing what to do |  |  |  |  |
| Fear of getting injured |  |  |  |  |
| Lack of company |  |  |  |  |
| Lack of support |  |  |  |  |
| Trouble with the work schedule |  |  |  |  |
| Lack of free time |  |  |  |  |
| Lack of conditions to exercise (weather, location, instruments…) |  |  |  |  |
| Other. Specify: |  |  |  |  |
